# Chimeric antigen receptors containing the OX40 signalling domain enhance the persistence of T cells even under repeated stimulation with multiple myeloma target cells

**DOI:** 10.1186/s13045-022-01244-0

**Published:** 2022-04-01

**Authors:** Jingwen Tan, Yujie Jia, Meixia Zhou, Chengcheng Fu, Israth Jahan Tuhin, Jing Ye, Masuma Akter Monty, Nan Xu, Liqing Kang, Minghao Li, Jiaqi Shao, Xiaoyan Fang, Hongjia Zhu, Lingzhi Yan, Changju Qu, Shengli Xue, Zhengming Jin, Suning Chen, Haiwen Huang, Yang Xu, Jia Chen, Miao Miao, Xiaowen Tang, Caixia Li, Zhiqiang Yan, Depei Wu, Lei Yu

**Affiliations:** 1 Institute of Biomedical Engineering and Technology, Shanghai Engineering Research Center of Molecular Therapeutics and New Drug Development, School of Chemistry and Molecular Engineering, East China Normal University, Shanghai, 200062 People’s Republic of China; 2 Shanghai Unicar Therapy Bio Medicine Technology Co Ltd., Shanghai, 201203 People’s Republic of China; 3 Jiangsu Institute of Hematology, National Clinical Research Center for Hematologic Diseases, the First Affiliated Hospital of Soochow University, Institute of Blood and Marrow Transplantation, Collaborative Innovation Center of Hematology, Soochow University, Suzhou, 215006 People’s Republic of China

**Keywords:** Chimeric antigen receptor T cells, Multiple myeloma, Persistence, Costimulatory molecules, OX40, CD28, 41BB

## Abstract

Persistence of CAR-T cell function is associated with relapse rate after CAR-T therapy, while co-stimulatory agents are highly concerned with the persistence of CAR-T cells. In this study, we designed and constructed a series of BCMA-targeting second-generation CAR constructs containing CD28, 41BB, and OX40 molecules, respectively, to identify the costimulatory domains most favorable for persistence. The results of routine in vitro studies showed that OX40-CAR-T and 41BB-CAR-T had similar antitumor effects and were superior to CD28-CAR-T in terms of proliferation and cytotoxicity. Although difficult to distinguish by conventional functional assays, OX40-CAR-T cells exhibited greater proliferation and enhanced immune memory than 41BB-CAR-T cells with the repeated stimulation assay by BCMA-expressing target cells. In vivo studies further demonstrated that OX40-CAR-T cells had stronger proliferative activity than 41BB-CAR-T cells, which was highly consistent with the in vitro antitumor activity and proliferation results. Our study provides for the first time a scientific basis for designing OX40-CAR-T cell therapy to improve relapse in patients with MM after CAR-T treatment.

**Supplementary Information:**

The online version contains supplementary material available at 10.1186/s13045-022-01244-0.

**To the editor,**

Multiple myeloma (MM) is the second most fatal hematologic malignancy, constituting 1–2% of neoplasms worldwide and being responsible for 2% of all cancer deaths [1]. Anti-BCMA-directed CAR-T cells treatment has achieved impressive response rate unfortunately most patients eventually relapse soon due to the poor persistence of CAR-T cells which closely related with different costimulatory molecules [2–5]; therefore, it is necessary to investigate the new costimulatory molecules to enhance this property of CAR-T cells.

Therefore, we constructed a serial of BCMA-targeted CARs containing 41BB, CD28, and OX40 co-stimulatory domain (Fig. 1A and Additional file 1: Fig. S1A), respectively, and investigated the effect on their duration of the antitumour properties. Firstly, the three groups of CAR-T cells showed comparable activation, differentiation, and apoptosis performance (Additional file 1: Fig. S1B–D and Additional file 2: Fig. S2A). However, the cytokine secretion experiment showed OX40-CAR-T cells were more prone to release the Th1 cytokines IFN-γ and TNF-α (Fig. 1B); while CD28-CAR-T cells tended to release the immunosuppressive cytokines IL-4 and IL-10 (Additional file 2: Fig. S2B and C). The exhausted markers of LAG-3, PD-1, Tim-3, and CTLA-4 inhibitory molecules of the cells were observed higher in the CD28-CAR-T cells than in the other two groups (Fig. 1C and Additional file 2: Fig S2D). With traditional experiment protocols [6], the OX40-CAR-T and 41BB-CAR-T cells showed equivalent proliferation and cytotoxicity profiles, but were significantly better than that of the CD28-CAR-T cells (Fig. 1D and E, P < 0.001 and P < 0.01, respectively). All the data indicated that the inducible co-stimulatory molecule of OX40 and 41BB might have better persistency than the primary co-stimulatory molecule of CD28 [7], whereas, the common evaluation methods could not distinguish the differences of the OX40 and 41BB of the BCMA-targeted CAR-T.

**Fig. 1:**
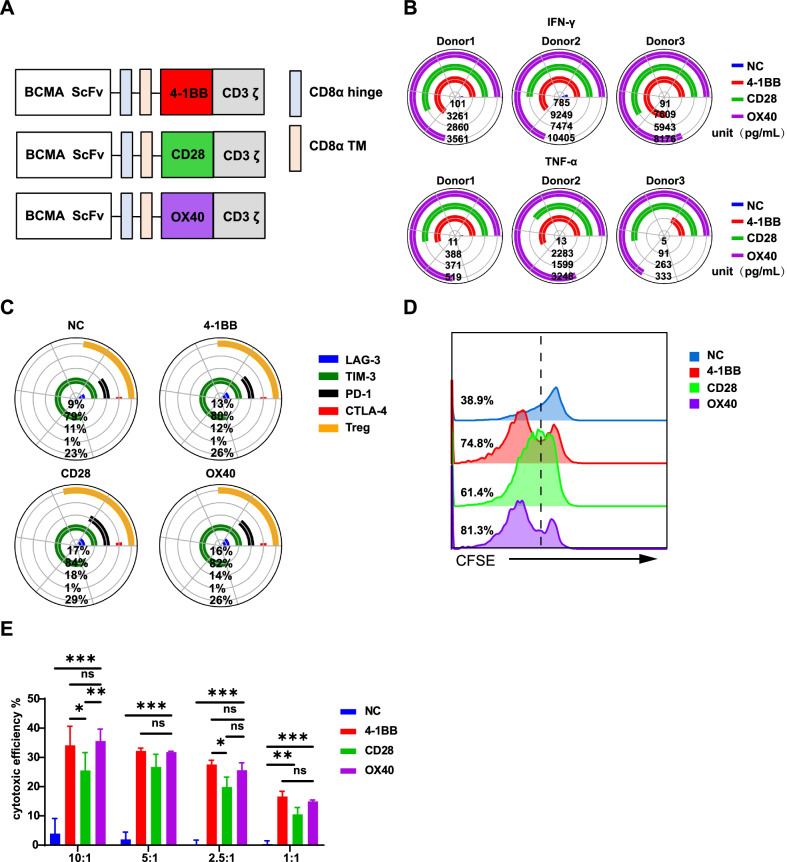
Characterizations of antitumour efficacy among CD28, OX40 and 41BB based CAR-T cells. **A** The structure of the BCMA-CAR consists of the same variable region of the BCMA single-chain antibody, the CD8 hinge and transmembrane regions, different costimulatory molecules (from 41BB, CD28, or OX40) and CD3ζ. **B** Coincubation of effector cells with target 8226 cells for 24 h at a ratio of 5:1 between D10 and D15 (different days were used in different donors) and the supernatant was collected. Cytokines were detected by a human Th1/Th2/Th17 kit using flow cytometry. The qualitative analysis of the expression of IFN-γ, TNF-α was performed with Phyton 3.7 using the Matplotlib package (https://matplotlib.org/) (*n* = 3 donors). **C** The expression of the exhaustion-related markers LAG-3, TIM-3, PD-1, and CTLA-4 on T cells expressing BCMA-CAR were measured on day 7 (D7) (*n* = 3 donors). Data were analyzed with FlowJo software, and graphs were plotted with Phyton 3.7 using the Matplotlib package (https://matplotlib.org/). **D** The Cell Trace TM CFSE Cell Proliferation Kit was used to detect cell proliferation. On D13, effector T cells were stained with CFSE (CFDA-SE) dye and incubated with target K562 (negative control) and 8226 cells at a ratio of 5:1. After 5 days of incubation, CFSE fluorescence intensity was detected by flow cytometry (K562 data not shown). **E** Effector T cells were incubated for 24 h with target K562 (negative control) and 8226 cells at E:T ratios of 10:1, 5:1, 2.5:1, and 1:1 on D13. Cytotoxicity was determined from the amount of released LDH in the culture supernatants using an LDH kit at a wavelength of 490 nm. The figure shows the result of effector T cells incubated with the 8226 target cells (*n* = 3, *P* < 0.001 and *P* < 0.01, error bars denote standard deviation)

To investigate the durability discrepancies between OX40 and 41BB co-stimulatory molecules of BCMA-targeted CAR-T, we designed a novel in vitro approach that imitates the killing process of tumor cells in the body by repeatedly stimulating CAR-T cells with target cells to induce their exhaustion. Surprisingly, we found that T effect memory (Tem) cells of BCMA-targeted OX40-CAR-T accounted for 60.0% (around two times) than that seen in the BCMA-targeted 41BB-CAR-T group (Fig. 2A and Additional file 3: Fig S3A), which is consistent with previous reported data of CD30-targeted OX40-CAR-T cells showed long-term immune memory [8]. And the percentage of CAR^+^ cells remarkably increased in the BCMA-targeted OX40-CAR-T cells group, reaching almost 80% (Fig. 2B), which indicated that in the case of consecutive antigen exposure, BCMA-targeted OX40-CAR-T cells proliferate rapidly. Finally, the data from in vitro culture experiment demonstrated that introducing OX40 as a costimulatory molecule had a crucial role in maintaining a high number of CAR^+^ cells (Fig. 2C and Additional file 2: Fig S2E) and reduced the incidence of loss of CAR^+^ cells (Additional file 2: Fig S2F).

**Fig. 2:**
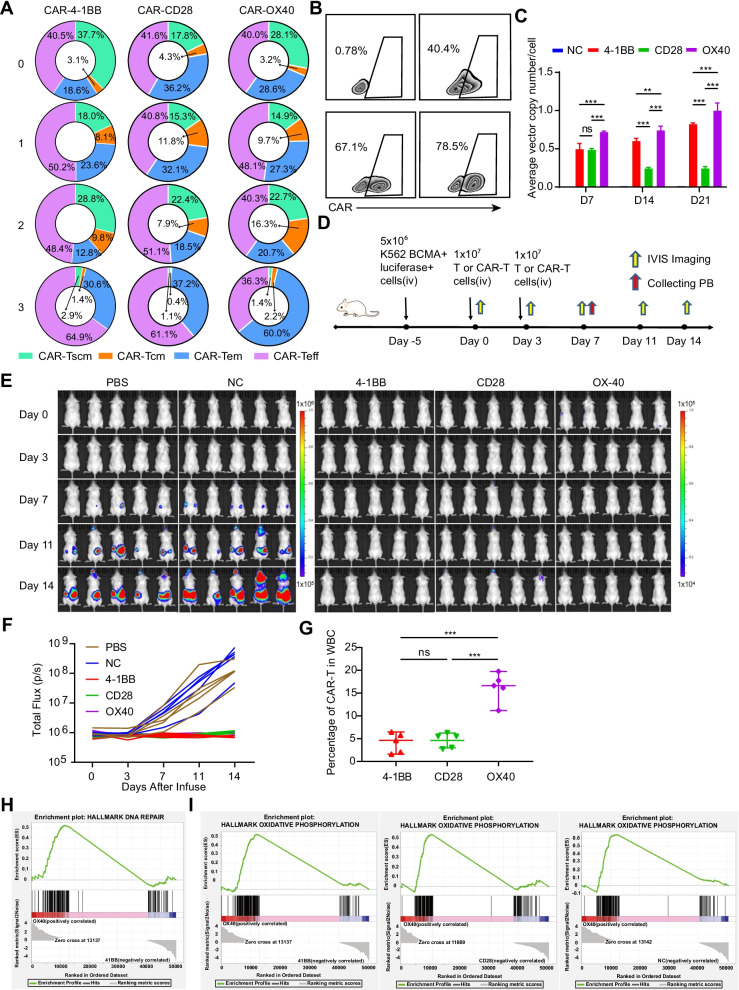
OX40-CAR-T cells are the least exhausted and have superior persistence. **A** Effector cells were subjected to three consecutive repeated stimulations with 8226 target cells (CAR^+^ cells and 8226 target cells at a ratio of 1:1; 3-day interval was used for each stimulation), changes in CAR^+^ cell subtypes were determined with flow cytometry (*n* = 3). **B** CAR^+^ cells from different experimental groups after repeated stimulation. As described in **A**, FlowJo was used for data analysis and presentation (*n* = 3). **C** The average copy number of the CAR gene in single cells was detected by qPCR technology on day 7 (D7), D14, and D21 after the cells were activated with anti-CD3/CD28 antibodies (*n* = 3, *P* < 0.001 and *P* < 0.01, respectively. error bars denote standard deviation). **D** Schematic outline of the mouse model experiment (*n* = 5). All the Methods and Materials were described in the Additional file 4. **E** Tumour progression was monitored by IVIS imaging. In order to scientifically show the small gap between different CAR-T, the scales are normalized for PBS and NC group with 1 × 10^5^ ~ 1 × 10^6^, and 4-1BB, CD28 and OX40 CAR-T group with 1 × 10^4^ ~ 1 × 10^5^. **F** Tumour progression was monitored by total flux of each mice. **G** The proportion of CAR-T in WBC (white blood cell) were detected from mice PB by flow cytometry on day 7. ***P ≤ 0.001, NS no significant. **H** Representative GSEA of DNA repair pathways and MSigDB hallmark gene set for two different BCMA-targeted CAR-T cells (OX40-CAR-T cells and 41BB-CAR-T cells). **I** Representative GSEA of the oxidative phosphorylation pathway and MSigDB hallmark gene sets for different BCMA-targeted CAR-T cells

We observed the similar phenomenon in our in vivo study with the repeatedly stimulating BCMA CAR-T (Fig. 2D). The more lower fluorescence intensity (Fig. 2E, F) reflected the superior efficacy of BCMA CAR-T, more importantly, 4-1BB and OX40 CAR-T cells seems to have a more lasting anti-tumour effect, compared to two mice of CD28 CAR-T are about to relapse(Fig. 2E). BCMA-targeted OX40-CAR-T cells could proliferate rapidly in vivo (Fig. 2G). This is consistent with the results of in vitro study.

To further explore the mechanism that OX40-CAR-T cells displayed unique persistence advantages, transcriptomic analysis of each group of BCMA-targeted CAR-T cells were performed and found that the increased gene expression profiles of proteins known to strengthen DNA repair (which enabled improved BCMA-targeted OX40-CAR-T survival activity) (Fig. 2H) and metabolism (which enabled enhanced proliferation and immune memory) (Fig. 2I and Additional file 3: Fig S3B) in the OX40-CAR-T group. At the same time, the enrichment of the TNF-α and IFN-γ signalling pathways explains the effective killing power of the BCMA-targeted OX40-CAR-T cells (Additional file 3: Fig S3C). These findings have not been reported elsewhere before.

In conclusion, our study demonstrated for the first time that OX40-mediated BCMA-targeted CAR-T showed more durable antitumor activity than 41BB-mediated CAR-T cells upon repeated stimulation of BCMA-expressing target cells. Our findings not only provide a scientific basis for designing novel BCMA-targeted CAR-T cells for MM to gain more durable anti MM activities, but also provide valuable data for improving the anti-tumor persistence and reducing recurrence after CAR-T cell therapy.

## Supplementary Information

**Additional file 1: Figure S1**. Evaluation of transduction efficiency, activation, differentiation abilities of BCMA-CAR-T cells.

**Additional file 2: Figure S2**. Comparison of apoptosis, cytokine inducing abilities and persistence of BCMA-CAR-T cells.

**Additional file 3: Figure S3**. Representative Flow cytometry analysis about CAR-T cells subtypes and Gene set enrichment analysis results.

**Additional file 4**. Detailed materials and methods as well as experimental procedures.

## Data Availability

All data generated or analyzed during this study are included in this published article (and its supplementary information files).

## Abbreviations

MM: Multiple myeloma
CAR: Chimeric antigen receptors
BCMA: B cell mature antigen
Tem: T effector memory cell
