# Supplementary material for: Chimeric antigen receptors containing the OX40 signalling domain enhance the persistence of T cells even under repeated stimulation with multiple myeloma target cells
Source: J Hematol Oncol. 2022 Apr 1;15:39. doi: 10.1186/s13045-022-01244-0 (PMC8974082; doi:10.1186/s13045-022-01244-0)
Supplement: Supplementary file 1 — Additional file 1: Figure S1. Evaluation of transduction efficiency, activation, differentiation abilities of BCMA-CAR-T cells. [file 13045_2022_1244_MOESM1_ESM.pdf]

Supplementary Figure 1

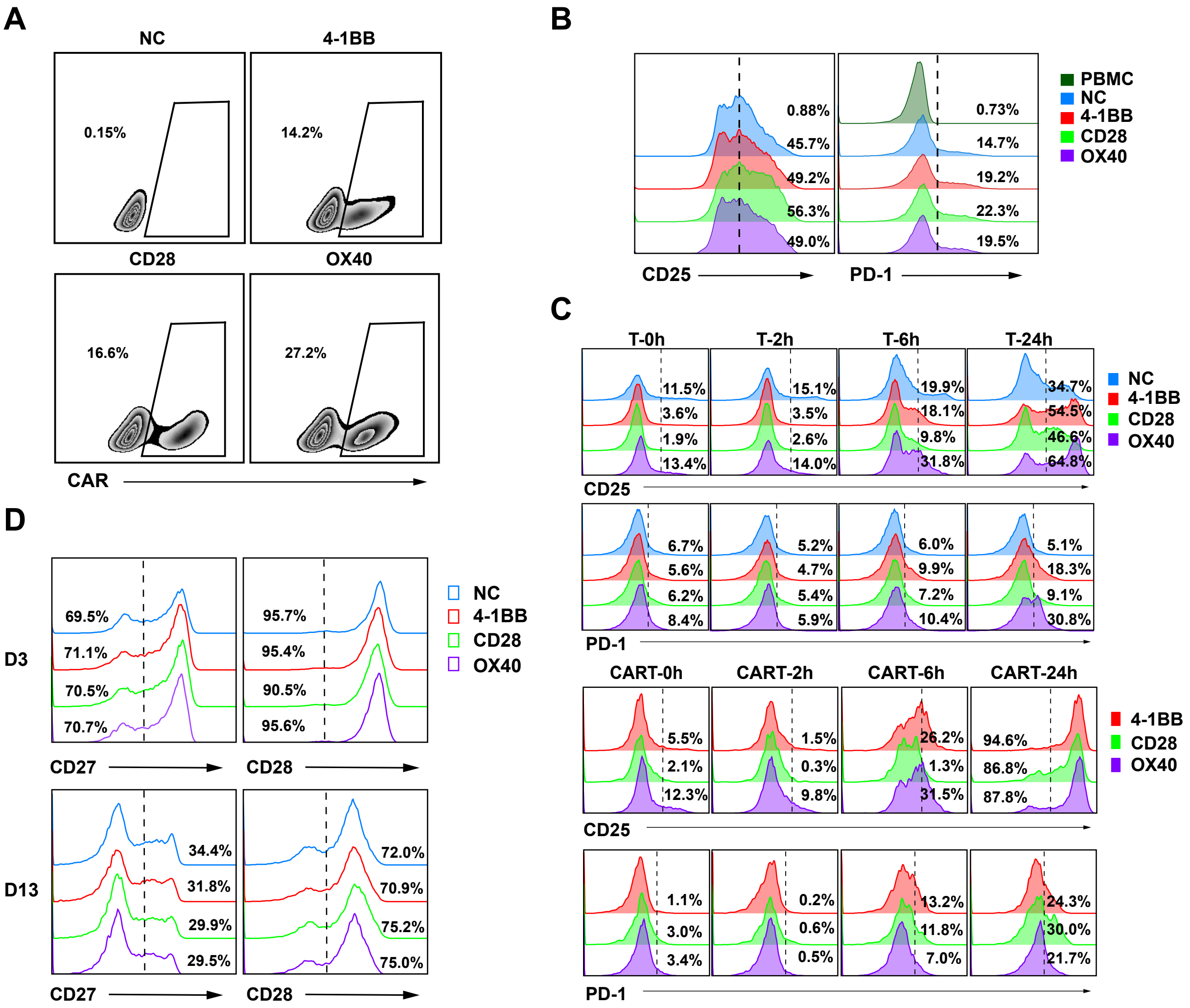

Supplementary Figure 1: Evaluation of transduction efficiency, activation, differentiation abilities of BCMA-CAR-T cells. A: Flow cytometry was used to determine the percentage of CAR+ cells on day 2 after lentivirus infection. Data were analysed and visualized by FlowJo. B: Activation was detected with flow cytometric identification of the surface expression of the activation markers CD25 and PD-1. T cells were stimulated with anti-CD3/CD28 antibodies on day 0 (D0), and then viral transfection of the T cells was performed. On D3, activation markers were detected. PBMCs were used as the non-stimulated group, and activation markers for this experimental group were gated on CD3. FlowJo was used for data analysis and visualization. C: Effector cells were incubated with target K562 (negative control) and 8226 cells at a ratio of 5:1, and samples were taken at 0 h, 2 h, 6 h, and 24 h to detect T cells and CAR-T cells by flow cytometry. The expression of CD25 and PD-1 was analysed using FlowJo. Data from the coincubation of effector cells and 8226 cells are shown. D: Expression of CD27 and CD28 in different experimental effector T cells was detected on D3 and D13 using flow cytometry, and data were analysed with FlowJo.
