# Supplementary material for: Chimeric antigen receptors containing the OX40 signalling domain enhance the persistence of T cells even under repeated stimulation with multiple myeloma target cells
Source: J Hematol Oncol. 2022 Apr 1;15:39. doi: 10.1186/s13045-022-01244-0 (PMC8974082; doi:10.1186/s13045-022-01244-0)
Supplement: Supplementary file 2 — Additional file 2: Figure S2. Comparison of apoptosis, cytokine inducing abilities and persistence of BCMA-CAR-T cells. [file 13045_2022_1244_MOESM2_ESM.pdf]

Supplementary Figure 2

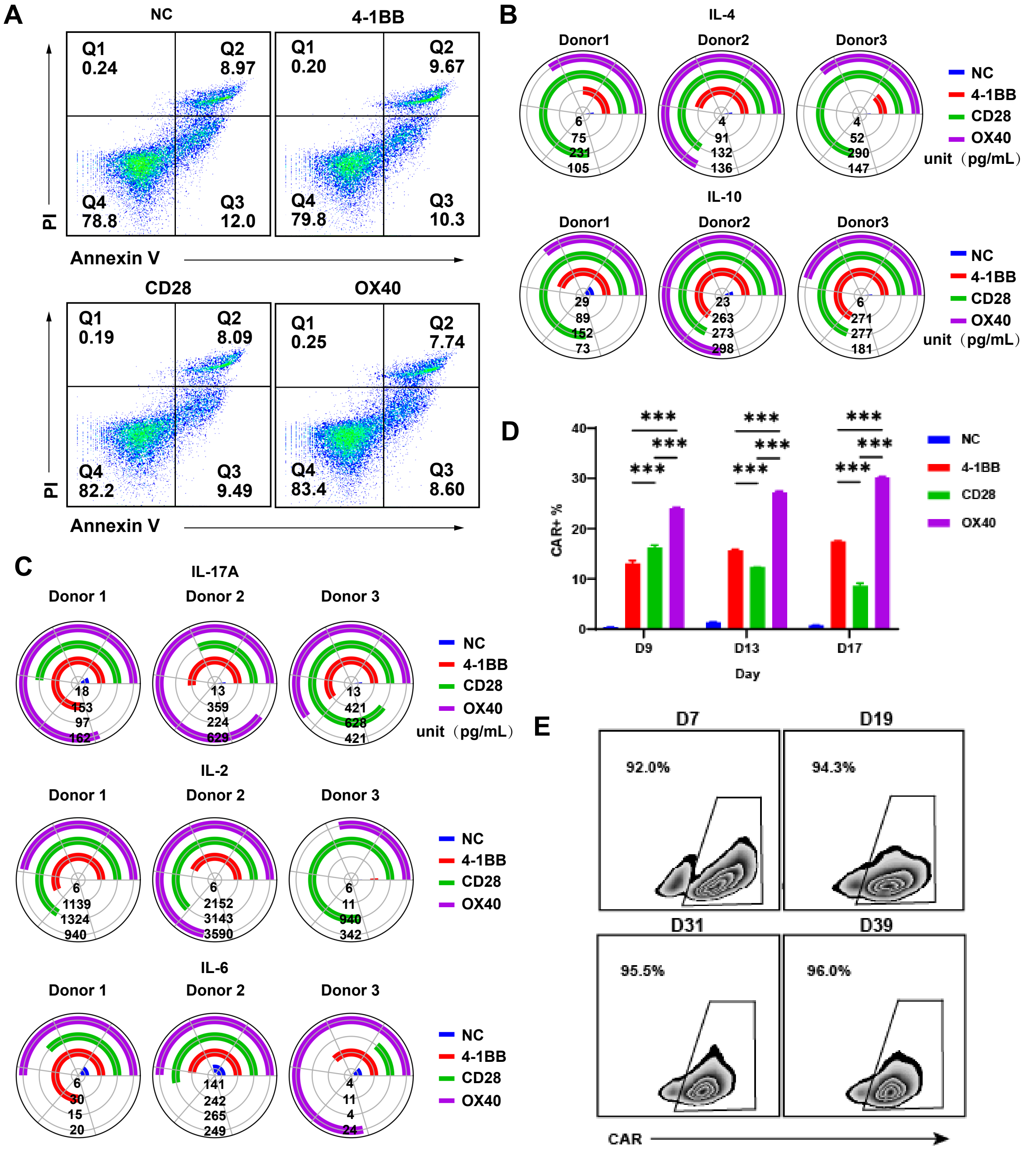

Supplementary Figure 2

Supplementary Figure 2: Comparison of apoptosis, cytokine inducing abilities and persistence of BCMA-CAR-T cells. A: Cell apoptosis was evaluated with an Annexin V-FITC cell apoptosis detection kit on D13. Data were analysed with FlowJo. B: Cytokines were detected by flow cytometry after coincubation of effector cells with target 8226 cells. The qualitative analysis of

the expression of IL-4 and IL-10 was performed with Phyton 3.7 using the Matplotlib package (https://matplotlib.org/)(n=3 donors). C: Cytokines were detected by flow cytometry after coincubation of effector cells with target 8226 cells. The qualitative analysis of the expression of IL-17A ,IL-2 and IL-6 was performed with Phyton 3.7 using the Matplotlib package (https://matplotlib.org/) (n=3 donors). D: The proportion of CAR+ cells was detected by flow cytometry on D9, D13, and D17 after the cells were activated with anti-CD3/CD28 antibodies (n=3, P<0.001, error bars denote standard deviation). E: Cells were stimulated with anti-CD3/CD28 antibodies. On D7, cells were sorted and identified by anti-BCMA-FITC antibodies and anti-FITC microbeads. Flow cytometry was used to detect CAR+ cells in the OX40-CAR-T cells, and the percentage of CAR+ cells was determined on D19, D31, and D39. Data were analyzed and visualized with FlowJo.
