# Supplementary material for: Chimeric antigen receptors containing the OX40 signalling domain enhance the persistence of T cells even under repeated stimulation with multiple myeloma target cells
Source: J Hematol Oncol. 2022 Apr 1;15:39. doi: 10.1186/s13045-022-01244-0 (PMC8974082; doi:10.1186/s13045-022-01244-0)
Supplement: Supplementary file 3 — Additional file 3: Figure S3. Representative Flow cytometry analysis about CAR-T cells subtypes and Gene set enrichment analysis results. [file 13045_2022_1244_MOESM3_ESM.pdf]

Supplementary Figure 3

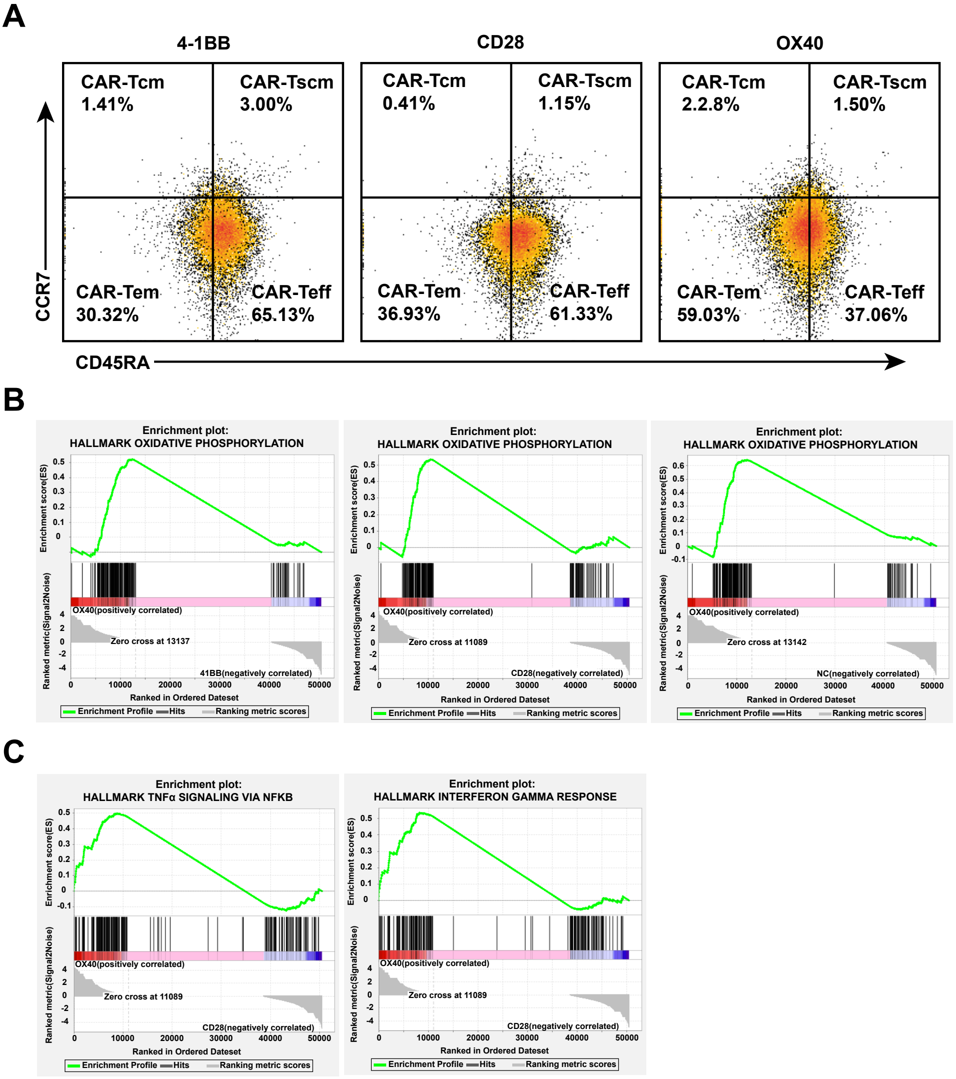

Supplementary Figure 3: Representative Flow cytometry analysis about CAR-T cells subtypes and Gene set enrichment analysis results. A: Effector cells were subjected to three consecutive repeated stimulations with 8226 target cells (CAR+ cells and 8226 target cells at a ratio of 1:1; a 3-day interval was used for each stimulation), CAR+ cell subtypes were determined with flow cytometry (n=3). B: GSEA of hypoxia, glycolysis, and fatty acid metabolism and MSigDB hallmark gene set for two different BCMA-targeted CAR-T cells (OX40-CAR-T cells and 41BB-CAR-T cells). C: Representative GSEA of TNF-α signalling via NFκB and the IFN-γ response and MSigDB hallmark gene sets for two different BCMA-targeted CAR-T cells(OX40-CAR-T cells and CD28-CAR-T cells).
