# Supplementary material for: Chimeric antigen receptors containing the OX40 signalling domain enhance the persistence of T cells even under repeated stimulation with multiple myeloma target cells
Source: J Hematol Oncol. 2022 Apr 1;15:39. doi: 10.1186/s13045-022-01244-0 (PMC8974082; doi:10.1186/s13045-022-01244-0)
Supplement: Supplementary file 4 — Additional file 4. Detailed materials and methods as well as experimental procedures. [file 13045_2022_1244_MOESM4_ESM.pdf]

Materials and Methods

Cell lines and culture conditions

The leukaemia cell line K562 (antigen-negative control) and the MM cell line 8226 were purchased from the American Tissue Culture Collection (ATCC; Manassas, VA, USA) and cultured in RPMI 1640 medium (Gibco, Grand Island, NY, USA) supplemented with 10% foetal bovine serum (Biological Industries, Kibbutz, BH, Israel) and 1% penicillin-streptomycin (Gibco). The HEK293T cell line was also purchased from ATCC and cultured in DMEM (Gibco) supplemented with 10% FBS (Biological Industries). Cells were maintained at 37°C and 5% CO_2 under fully humidified conditions throughout the experimental period. K562 BCMA cells for xenograft models were engineered to overexpress BCMA-luciferase by lentiviral infection.

Lentiviruses vector construct design and production

The lentiviruses encoding the three CAR constructs were generated by modifying a consecutive 2nd-generation CAR sequence in a frame directed 5′ end to the 3′, which consisted of anti-BCMA-scFv (C11D5.3), the hinge and transmembrane regions of the CD8α molecule, one of the three costimulatory domains (CD28, 41BB or OX40), and the CD3ζ signalling domain. These sequences were synthesized by Tsingke Biological Technology (Shanghai, China) and cloned using the pUT plasmid backbone (Unicar-Therapy Biomedicine Technology Co., Ltd., Shanghai, China). Replication-defective lentiviruses were packaged with transient transfection of HEK293T cells using these designed constructs.

CAR T cell preparation and expansion

Healthy donor-derived peripheral blood was collected, and mononuclear cells (PBMCs) were isolated with the Ficoll-Paque gradient centrifugation method using Human Mononuclear Cell Separation Medium (Oriental Hua Hui, Beijing, China). $CD4^+CD8^+$ T cells were enriched through positive selection using the magnetic bead cell separation method (Miltenyi Biotec, Bergisch Gladbach, Germany). Isolated cells were activated with anti-CD3/CD28 monoclonal antibodies (Miltenyi Biotec) for 18-24 h. This time was considered D0, and the rest of the time was calculated from this point. Then, the activated T cells were transduced with lentivirus (41BB-CAR, CD28-CAR and OX40-CAR) for 48 h. Furthermore, CAR^+ cells were sorted using FITC-conjugated anti-BCMA antibody (ACRO Biosystems, Beijing, China) and anti-FITC microbeads (Miltenyi Biotec) on day 7. Cells were maintained in AIM-V medium (Gibco) supplemented with 1,000 IU/mL recombinant human interleukin-2 (IL-2; Peprotech, Rocky Hill, NJ, USA) and 5% FBS (Gibco). Fresh complete medium was added every 2-3 days, and the cells were maintained at a density of 5×10^5 - 1×10^6/ml.

Flowcytometry

All samples were performed on an Attune NxT flow cytometer (Thermo Fisher, Waltham, USA), and data were analysed with FlowJo V10 software (TreeStar, San Carlos, CA, USA). A PE-conjugated anti-human CD269 (BCMA) antibody (Biolegend, San Diego, CA, USA) was used to examine BCMA antigen expression. The expression of the BCMA-CAR was detected with the Biotinylated Human BCMA Protein (ACRO), followed by APC-conjugated streptavidin (BD Pharmingen,

San Diego, CA, USA) or a FITC-labelled Human BCMA protein Fc Tag (ACRO) was used. CD4/CD8 subsets were quantified by AlexaFluor700-conjugated anti-CD4 monoclonal antibody (eBioscience, San Diego, CA) and APC/Cyanine7-conjugated anti-human CD8 antibody (BioLegend) staining. T cell phenotypes were identified with FITC-conjugated anti-CD223 (LAG-3) monoclonal antibody (eBioscience), PE-conjugated mouse anti-human TIM-3 (CD366) antibody (BD Pharmingen), PE-Cyanine7-conjugated CD152 (CTLA-4) monoclonal antibody (eBioscience), PerCP/Cyanine5.5-conjugated anti-human CD279 (PD-1) antibody, PE-Cyanine7-conjugated anti-CD127 monoclonal antibody (eBioscience), PerCP/Cyanine5.5-conjugated anti-human CD45RA antibody (Biolegend), FITC-conjugated anti-human CD25 antibody (BioLegend), and PE-conjugated anti-human CD197 (CCR7) antibody (BioLegend) staining. In addition, PE-conjugated anti-human CD27 antibody (BioLegend) and APC-conjugated anti-human CD28 antibody (BioLegend) staining were used to analyse senescent T cells. To evaluate the degree of apoptosis, cells were stained with Annexin V-FITC and propidium iodide (Beyotime, Shanghai, China). For the flow cytometry assays, all cells were harvested and washed three times with FACS wash buffer (1X PBS containing 0.5% BSA and 0.03% sodium azide), and cells were stained according to the manufacturer's instructions.

Cell proliferation assay

A T cell proliferation assay was performed according to the CFSE cell staining assay method using the Cell TraceTM CFSE Cell Proliferation Kit (Invitrogen, Carlsbad,

CA, USA). CAR T cells were labelled with 2.5 μM CFSE and cocultured with K562 and 8226 target cells. Before coculture, target cells were treated with mitomycin (Selleckche, USA) to stop cell division. Non-transduced T cells (NC) were used as a negative control. Cells were plated in a 24-well plate at an effector-to-target (E: T) ratio of 5:1 and cocultured for 5 days in a total volume of 500 μl of AIM-V medium (Gibco) supplemented with 4% FBS (Gibco). Proliferation was determined from the CFSE dilution after 3-5 days by flow cytometry using an Attune NxT flow cytometer (Thermo Fisher), and data were analysed with FlowJo V10 software (TreeStar).

Cytotoxicity assay

To measure cytotoxicity, CAR T cells were plated with target K562 and 8226 cells at E:T ratios of 10:1, 5:1, 2.5:1, and 1:1. Cells were plated in a 96-well plate in triplicate wells in a total volume of 100 μl of AIM-V medium (Gibco) supplemented with 4% FBS (Gibco). Cocultured cells were incubated for 18-24 h. Supernatants from each well were collected, and the cytotoxicity was determined via quantitation of lactate dehydrogenase using a Cytotoxicity Detection Kit (Promega, Madison, WI, USA) according to the manufacturer's protocol. Absorbance was measured at 490 nm using a Multiskan GO wavelength reader (Thermo Scientific). Tumour cell (target cell) lysis was calculated with the following formula: % lysis = (experimental LDH release – spontaneous LDH release) / (maximum LDH release − spontaneous LDH release) × 100%.

Cytokine detection

Cells were plated at an E:T ratio of 5:1 and cocultured for 24 h. The supernatant was collected, and cytokines (IL-2, IL-4, IL-6, IL-10, IFN-γ, TNF-α, and IL-17A) were measured with a Th1/Th2 Cytometric Bead Array (CBA) Kit II (BD Bioscience) following the manufacturer's instructions. Data were analysed with LEGENDplex v8.0 (Biolegend), and cytokine concentrations were calculated and determined from standard curves.

Tumour cell-mediated activation of CAR T cells

T cell activation was evaluated by measuring CD25 and PD-1 (CD279) expression on 41BB-CAR-T cells, CD28-CAR-T cells and OX40-CAR-T cells. Effector cells were cocultured with or without target cells, and expression was measured. Non-transduced T cells were used as negative controls (NC), and 8226 cells were used as target cells. Cells were plated in a 24-well plate at a 5:1 E:T ratio. After coculture for 0, 2, 6 and 24 h, the cells were harvested and washed twice with PBS. Next, the cells were labelled with APC/Cyanine7-conjugated anti-human CD3 antibody (BioLegend), APC-conjugated anti-human CD25 antibody (BioLegend) or FITC-conjugated anti-human CD25 antibody (BioLegend) and PE/Cyanine7-conjugated anti-human CD279 (PD-1) antibody (BioLegend) or APC-conjugated anti-human CD279 (PD-1) antibody (BioLegend) at 4°C for 20 minutes in the dark. The BCMA-CAR was detected with a FITC-labelled human BCMA protein Fc tag (ACRO). Activation marker expression on T cells and CAR-T cells was detected by flow cytometry (Thermo Fisher).

Gene expression analysis by qPCR

Genomic DNA was extracted from cultured CAR-T cells using a DNA extraction kit (Generay Biotech, Shanghai, China). For quantitative PCR (qPCR), we used the SYBR green real-time PCR method. The primer sequences used for measuring CAR copies were 5´-GAGCCTGGTGATTACCCTGTATTG-3´ (forward) and 5´-TGGTTCTGGCCCTGCTGATA-3´ (reverse).

RNA sequencing, gene expression analysis and GSEA

Total RNA was extracted using TRIzol Reagent (Life Technologies, Carlsbad, CA, USA) following the manufacturer’s instructions, and the RIN number was calculated with an Agilent Bioanalyzer 2100 (Agilent Technologies, Santa Clara, CA, USA) to inspect RNA integrity. Qualified total RNA was further purified with an RNAClean XP Kit (Beckman Coulter, Inc. Kraemer Boulevard Brea, CA, USA) and RNase-Free DNase Set (QIAGEN, GmBH, Germany). Differentially expressed genes were selected with criteria of fold change $\geqslant$ 2 and p $\leqslant$ 0.05. Cluster analysis and principal component analysis were conducted with Partek default settings. Heatmaps were generated with the Partek Genomics Suite using normalized data standardized on the average. Gene set enrichment analysis (GSEA) was performed using Broad Institute software (http://www.broadinstitute.org/gsea/index.jsp). The GSEA used gene sets from the Molecular Signature Database v4.1.0. The normalized enrichment score (NES) was calculated by permutation testing.

Mouse Xenograft models

All the animal experiments were performed with the approval of the Institutional Animal Care and Use Committee of East China Normal University. 6-week-old

NOD/ShiLtJGpt-Prkdc^{em26}Il2rg^{em26}/Gpt (NCG) mice (Jiangsu GemPharmatech Co, Ltd, China) were injected 5 × 10^6 K562 BCMA$^+$ luciferase$^+$ cells through tail vein to built mouse xenograft models on day -5. Then, 1 × 10^7 4-1BB-CAR-T, CD28-CAR-T, OX40-CAR-T, T cell or PBS were infused into mice through tail vein on day 0 and day 3. Before the first infusion, CAR-T cells were subjected to two consecutive repeated stimulations with K562 BCMA$^+$ luciferase$^+$ cells (Effector cells and target cells at a ratio of 1:1). Imaging of mice was acquired with a Xenogen-IVIS system (LICOR Biosciences, Lincoln, NE, USA) on day 0 to quantify engraftment and tumour progression was measured twice per week. And Living Image software (IVIS Lumina Series, PerkinElmer, Waltham, MA, USA) was applied to analyze the bioluminescent signals. The PB (peripheral blood) of mice was collected on days 7. PB of day 7 was assessed by flow cytometry to detect CAR-T percentage of WBC (white blood cell).

Statistical analysis

Data were analysed with GraphPad Prism 8.0 and Python 3.7. Statistical significance was calculated using one-way and two-way ANOVA. Experimental replicates were performed in triplicate. All the data are presented as the mean ± standard deviation (SD). Error bars denote standard deviation. $P<0.05$ was considered to indicate significance and is presented as an asterisk in the figures.
